# Renal and hepatic artery embolization with Pickering gel emulsion of lipiodol in rabbit

**DOI:** 10.1186/s12885-022-10337-5

**Published:** 2022-12-12

**Authors:** Hongsen Zhang, Yanqiao Ren, Han Li, Chuansheng Zheng, Kun Qian

**Affiliations:** 1 Department of Radiology, Union Hospital, Tongji Medical College, Huazhong University of Science and Technology, 430022 Wuhan, China; 2 Hubei Province Key Laboratory of Molecular Imaging, 430022 Wuhan, China; 3 National Engineering Research Center for Nanomedicine, Key Laboratory of Molecular Biophysics of Ministry of Education, Hubei Key Laboratory of Bioinorganic Chemistry and Materia Medica, College of Life Science and Technology, Huazhong University of Science and Technology, 1037 Luoyu Road, 430074 Wuhan City, China; 4 The GBA National Institute for Nanotechnology Innovation, 136 Kaiyuan Avenue, Guangzhou, PR China

**Keywords:** Pickering gel emulsion of lipiodol, Embolic material, Renal artery, VX2 tumor, Transcatheter embolization

## Abstract

**Objective:**

This research aimed to evaluate the feasibility of a novel liquid embolic agent Pickering gel emulsion of lipiodol (PGEL) for renal and hepatic artery embolization in the rabbit experimental model.

**Methods:**

Embolization was performed in the right renal artery of 24 adult New Zealand White rabbits and 24 VX2 tumors in the left liver lobe. The rabbits were randomly allocated to four treatment groups (*n* = 6 per group): (A) normal saline (NS), (B) lipiodol, (C) 180–300 μm polyvinyl alcohol (PVA), and (D) PGEL.

**Results:**

Renal artery embolization in normal rabbits and transarterial embolization (TAE) in VX2 tumor-bearing rabbits indicated that PGEL achieved a better embolization effect for a longer time than lipiodol and PVA. The tumor growth ratio of the PGEL group was significantly lower than that of the NS, lipiodol, and PVA groups at 3 (*P* < 0.001) and 7 (*P* < 0.001) days after embolization. In addition, hematoxylin and eosin and immunohistochemical staining revealed that the tumor necrosis ratio was higher in the PGEL group than in the NS, lipiodol, and PVA groups (*P* < 0.01), and the expression levels of HIF-1α, VEGF, and CD31 decreased after PGEL embolization compared with the lipiodol and PVA treatments.

**Conclusion:**

PGEL is an effective embolic material that provides immediate and total occlusion of the renal artery and may be a potential therapeutic embolic agent for TAE of HCC.

**Supplementary Information:**

The online version contains supplementary material available at 10.1186/s12885-022-10337-5.

## Introduction

Hepatocellular carcinoma (HCC) is one of the most common malignant tumors worldwide. Curative treatment for HCC is not feasible because the majority of patients are already in the advanced stage of the disease at the time of diagnosis [1]. Transarterial embolization (TAE) and transarterial chemoembolization (TACE) are considered the most effective palliative treatments for HCC [2]. Embolic materials are indispensable for both TAE and TACE. Thus, various embolic materials have been developed and applied [3, 4]. Although no “one-size-fits-all” material exists for embolization, an ideal embolic material must satisfy various requirements, including good biocompatibility, easy delivery, and visibility and traceability on common imaging modalities [5, 6].

Lipiodol, as a radio-opacity agent, with retention in the tumor, has been widely used in conventional TACE for HCC since the early 1980s. The radio-opacity of lipiodol helps to monitor treatment delivery, with retention of lipiodol serving as an imaging biomarker for tumor response. However, its lack of capability for drug-controlled release and its low mechanical strength lead to recanalization of tumor arteries [7, 8], limiting the embolic efficacy of lipiodol. Other commercially available embolic materials, such as Gelfoam particles, polyvinyl alcohol (PVA) microspheres, and drug-eluting beads, have some limitations, including nonradiopacity, short tumor penetration, ease of aggregation, and unfavorable intraoperative and postoperative computed tomography (CT) evaluation [9–11].

Our previous studies [12, 13] revealed that poly (N-isopropyl acrylamide-co-butyl methyl acrylate) (PIB) nanogels could be used for HCC embolization through a thermosensitive sol-gel phase transition, and their ability to achieve artery-casting embolization has been demonstrated. However, PIB nanogels are not radiopaque. Therefore, they are mixed with a contrast agent for embolization of tumor donor arteries. The contrast agent may affect the thermosensitive property of the PIB nanogel, which may increase the risk posed by the operation.

In the previous study, the deformable hairy p (N-isopropylacrylamide-acrylic acid) nanogels with a core-shell structure (hPNA) and the hPNA emulsion (6.0 wt% hPNA as the water phase; lipiodol content of 30%) was synthesized [14]. In order to further improve its properties, this study presented a novel liquid embolic agent Pickering gel emulsion of lipiodol (PGEL). The prepared radiopaque embolic formulations with low viscosity can be easily injected through a microcatheter, and exhibit a sol-to-gel phase behavior of PIB nanogels. It is expected that the location and distribution of PGEL can be fed back in real-time under fluoroscopy/CT, and peripheral arterial embolization of tumors can also be achieved. The injectability and blood-vessel-embolic and angiographic abilities of PGEL after intra-arterial injection into renal arteries and arteries supplying the VX2 tumor were examined using CT images and excised kidney and tumor tissues.

## Materials and methods

### Materials and grouping

The embolic agent PGEL, a thermosensitive Pickering gel emulsion with highly dispersive stability, was fabricated by adsorption of deformable PIB nanogels on the oil (lipiodol)-water interface at the National Engineering Research Center for Nanomedicine, College of Life Science and Technology, Huazhong University of Science and Technology (Wuhan, China). We created the hPNA emulsion with 6.0 wt% hPNA as the water phase. The lipiodol content was 40% for better drug loading and a higher X-ray shielding ability, and we synthesized PGEL by two-step seed emulsion polymerization (an oil-in-water emulsion).

Adult New Zealand White rabbits of either gender (ca. 3.0 kg body weight) were provided by the Laboratory Animal Research Center. All animal experiments were approved by the Ethics Committee of Tongji Medical College, Huazhong University of Science and Technology (IACUC: 604). All experiments were performed in accordance with the relevant guidelines and regulations. The experiments also complied with the ARRIVE guidelines.

48 rabbits, including 24 normal rabbits for right renal artery embolization and the rest bearing VX2 tumors for hepatic artery embolization, were used to evaluate the injectability and blood-vessel-embolic and angiographic abilities of PGEL. PGEL, lipiodol, and PVA particles were used to embolize renal or hepatic arteries (*n* = 6 per group), and normal saline (NS) infusion served as the blank control group (*n* = 6).

24 rabbits were implanted with VX2 tumors in the left lobe of the liver following a previously described method [12]. Briefly, VX2 tumors were first extracted from carrier rabbits, sectioned into 1-mm^3^ slices under sterile conditions, and stored in physiological saline on ice. The left liver lobe of the recipient rabbit was then exposed via an abdominal midline incision, and a small fragment of the VX2 tumor was inoculated into the left lobe of the liver. A small piece of gelatin sponge was placed into the liver wound and the abdomen was closed in layers. The tumors were then allowed to grow for 14 days before TAE.

### Transarterial embolization of the renal artery

24 normal rabbits were given general anesthesia with pentobarbital sodium solution (2 wt%, 30 mg/kg) via the auricular vein after 12-h of fasting and water deprivation. After 5–10 min, the rabbits were fixed in the supine position. The right groin skin was incised, the common femoral artery was surgically exposed, and a 4-F sheath (Terumo, Tokyo, Japan) was placed. Under fluoroscopic guidance, the right renal artery was catheterized with a 4-F Cobra catheter (Terumo, Tokyo, Japan), and arteriography was performed by injecting a contrast agent (Omnipaque, 300 mg I/mL) at a rate of 0.5 mL/s. Afterward, PGEL, lipiodol, or PVA (Ivalon, 280–350 μm) was injected at a rate of 0.4 mL/s. Saline was infused as a control. The X-ray visibility of PEGL was evaluated by renal artery imaging using a spiral CT scanner (Aquilion ONE, Toshiba, Japan) at specified time intervals, while the other three groups were used as references. 12 weeks after the intervention, the four groups of rabbits were euthanized. Vital organs, including the heart, liver, spleen, lungs, and left kidney, were analyzed by hematoxylin and eosin (H&E) staining.

After PGEL was injected into the renal artery, the rabbits were sacrificed immediately, and the embolized kidneys were removed for transmission electron microscope (TEM) staining. Targeted fresh renal tissues were selected to minimize mechanical damage, such as pulling, contusion, and extrusion. A sharp blade was used to cut and harvest fresh tissue blocks within 1–3 min. Before sampling, Petri dishes with a TEM fixative solution were prepared in advance. Small tissue blocks were removed from the animal body, immediately placed into Petri dishes, and then cut into small pieces (1 × 1 × 1 mm) in the fixative. The tissue blocks were processed and observed with TEM, and images were captured with a digital camera.

### Transcatheter embolization of intrahepatic VX2 tumors

As with rabbit renal arteriography, catheterization of the celiac axis artery was achieved via a 4-F Cobra catheter (Terumo, Tokyo, Japan) and the VX2 tumor and its supplying artery were identified by angiography. The tumor donor artery was selectively catheterized with a 2.7-F microcatheter (Terumo, Tokyo, Japan) for embolization (PGEL or lipiodol or PVA) or perfusion (saline). The angiographic embolization endpoint was complete flow stasis, defined as no contrast delivery visible in the tumoral and peritumoral vessels while maintaining patency of the feeding hepatic artery.

### Follow-up CT angiography and histopathology

Pretreatment and 1, 3, and 7 days post-treatment contrast-enhanced CT were performed to assess the embolization effect. The rabbits were scanned by a 320-row spiral CT scanner (Aquilion ONE, Toshiba, Japan) with intravenous contrast agents using the following parameters: 80 kV, 60 mA, 1-mm slice thickness, field of view (78.92 × 78.92 mm), and effective pixel size 50 μm. Approximately 3.0 mL of the contrast agent iohexol (Omnipaque, 300 mg I/mL) and 10 mL of NS were injected for multiphase CT enhancement scans using a high-pressure syringe (Stellant Meorao) at a rate of 0.5 mL/s after tumor localization via a CT plain scan. The CT data were analyzed using Amira (4.1.2) software. The tumor growth ratio (GR) was calculated using the following equation: GR = a_2_b_2_^2^/a_1_b_1_^2^, where a_1_ and a_2_ are the maximum diameter of the tumor before and after TAE, respectively, and b_1_ and b_2_ are the minimal diameter of the tumor before and after TAE, respectively [15].

The rabbits were euthanized under deep anesthesia 7 days after TAE and saline infusion. Tumor and peritumoral normal tissue samples were harvested and fixed in 4% paraformaldehyde in phosphate-buffered saline, embedded in paraffin, and sectioned into 5-mm sections for subsequent histopathological staining preparations: (a) H&E staining was used to identify tumor histopathological changes and calculate the tumor necrosis ratio (TNR); (b) tumor apoptosis was determined by the terminal deoxynucleotidyl transferase biotin-dUTP nick end labeling (TUNEL) assay using an Apoptosis detection kit (R&D Systems, Minneapolis, MN, USA) according to manufacturer’s protocol; (c) different primary antibodies, including anti-Ki67 (dilution, 1:200; Abcam, USA), anti-VEGF (dilution, 1:150; Millipore, Billerica, MA), anti-CD31 (dilution, 1:50; Dako, Copenhagen, Denmark), and anti-HIF-1α (dilution, 1:100; Thermo, IL, USA) proteins for immunohistochemical (IHC) staining were used to identify angiogenesis and hypoxia signaling pathways within the tumor microenvironment.

Whole-section digital histological scans were acquired with a Pannoramic MIDI II scanner (3DHISTECH; Budapest, Hungary) yielding high-resolution image acquisition. TNR was calculated based on the percentage of the necrotic area to the total tumor area, which was calculated from the H&E staining sections as follows: TNR = N/(N + T), where N and T represent necrotic and non-necrotic areas of the tumor, respectively [16, 17]. The relative signal intensity was quantified by densitometry using Image-Pro Plus 6.0 software (Media Cybernetics; Silver Spring, MD) and the integrated optical density (IOD) was used to quantify the relative expression levels of TUNEL, Ki67, CD31, VEGF, and HIF-1α. The IOD of the fluorescence or number of positive cells per 100 µm^2^ was evaluated throughout the area from six fields of each sample slice.

Liver and kidney functions were evaluated using a biochemical autoanalyzer (Model DXC 8000; Beckman Coulter Diagnostics, Brea, CA, USA). After TAE and saline infusion, 2 mL of peripheral blood was collected at 1, 3, and 7 days. Plasma alanine aminotransferase (ALT), aspartate aminotransferase (AST), blood urea nitrogen (BUN), and serum creatinine (CRE) were determined. In addition, rabbit hearts, livers, spleens, lungs, and kidneys were harvested 7 days after TAE and saline infusion and then fixed in 4% paraformaldehyde in phosphate-buffered saline, embedded in paraffin, and sectioned into 5-mm sections for H&E staining.

#### Statistical analysis

All data are expressed as the mean ± standard deviation (SD). Statistical analysis was performed using Prism 5.0 (GraphPad Software; La Jolla, CA). Differences between treatment groups were compared with independent-samples t-test, and *P* < 0.05 was considered statistically significant.

## Results

### Characterization of PGEL

**Fig. 1:**
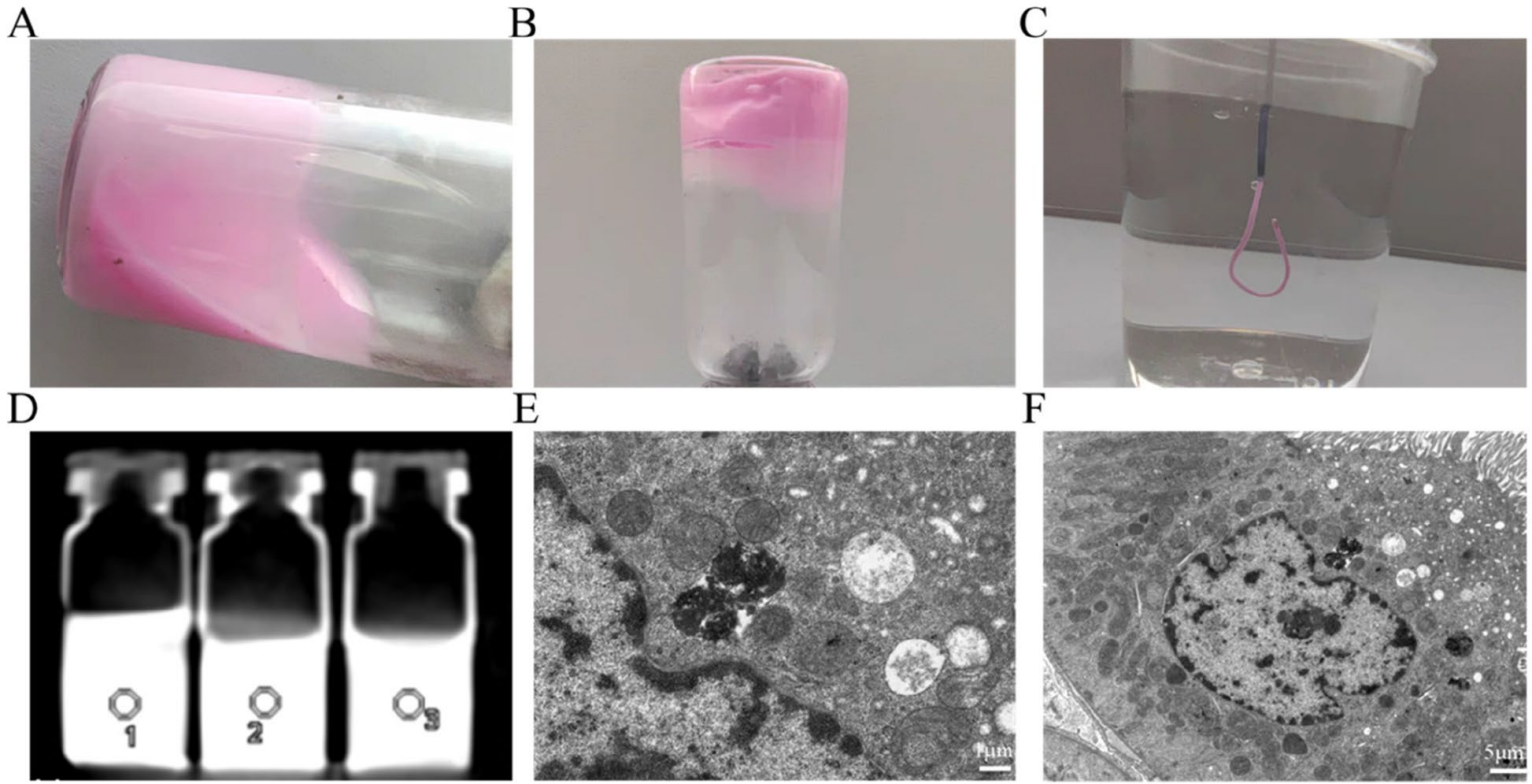
Properties of PGEL. **A** PGEL is a flowable solution at low temperature and **B** becomes a solid gel at high temperature (> 36 °C). **C** PGEL is injected into 37 °C water through a 2.7 F microcatheter and rapidly solidifies as a gel. **D** Comparison of X-ray attenuation of PGEL with Iomeprol and Omnipaque (from left to right). **E**, **F** TEM images of renal artery embolization with PGEL at different scales. PGEL is seen in the renal arteries and glomeruli (black particles are PGEL).

PGEL underwent a sol-gel transition at its lower critical solution temperature of 36.5 °C (Fig. 1A and B), and it turned into a gel within 3 s when injected into 37 °C water through a 2.7 F microcatheter (Fig. 1C). The X-ray attenuation ability of PGEL was compared with commercial iodinated X-ray contrast agents (Iomeprol and Omnipaque) using a CT scanner to explore the radiopacity of PGEL (Fig. 1D). As a reference, the CT value of Iomeprol solution (400 mg I/ml) was 5530.6 ± 40.2 HU. The CT value of PGEL (4461.9 ± 69.6 HU) was lower than that of Iomeprol solution but higher than that of 300 mg I/ml Omnipaque solution (3632.4 ± 54.7 HU). In addition, because the CT value of bone is approximately 1,000 HU, contrasts with a CT value higher than 1,000 HU are distinguished under clinical imaging modalities, which were observed in the subsequent in vivo assessments. TEM images of renal artery embolization with PGEL at different scales are shown in Fig. 1E F.

### Evaluation of the visibility of PGEL in normal rabbits

**Fig. 2:**
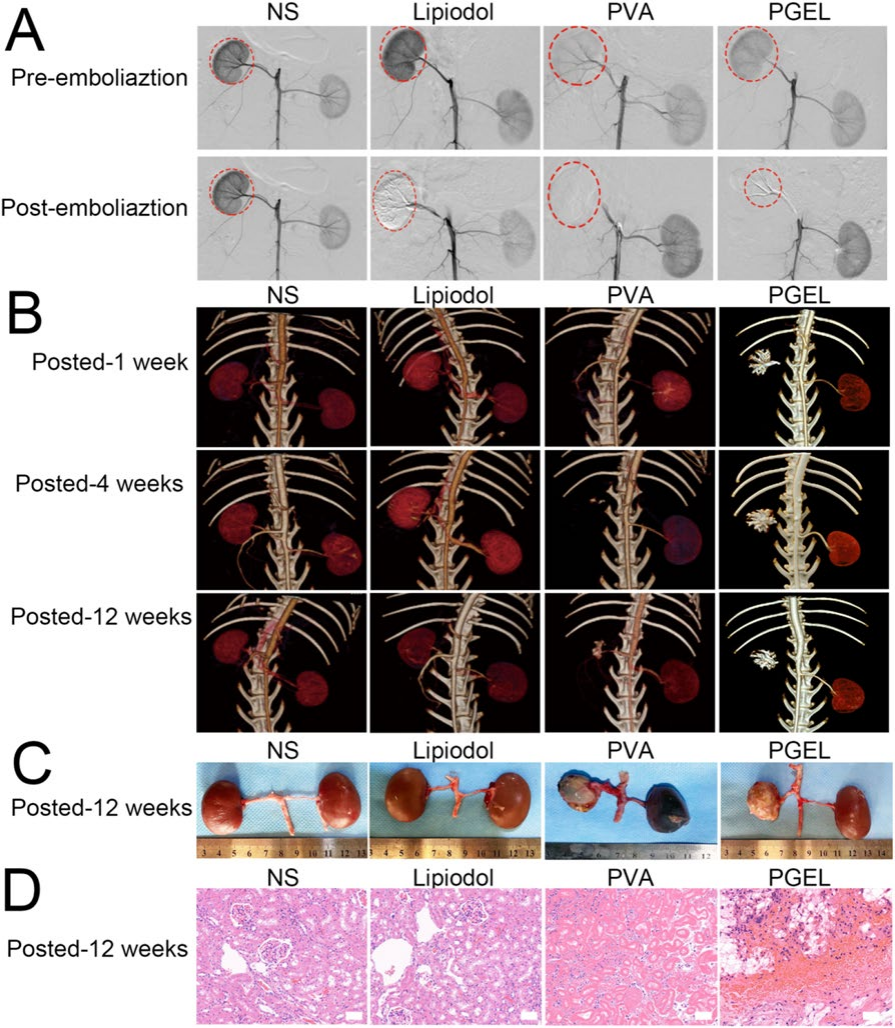
Comparison of renal artery embolization with normal saline (NS), lipiodol, PVA, and PGEL groups in normal rabbits. **A** Digital subtraction angiography (DSA) images of rabbit kidneys before and after embolization. Iohexol (350 mg I/mL) was used as a contrast agent for DSA. **B** Three-dimensional reconstruction images of X-ray computed tomography (CT) at 1, 4, and 12 weeks after embolization. **C** Gross photographs of rabbit kidneys at 12 weeks after embolization. **D** Kidney sections obtained at 12 weeks after embolization were stained with hematoxylin and eosin (H&E) and imaged at ×200 magnification (the scale bar represents 50 μm). PGEL is marked by yellow particles (indicated by red arrows)

PGEL was successfully injected into the renal arteries of normal rabbits without mixing with contrast agents and without clogging. As shown in Fig. 2A, PGEL exhibited angiographic abilities similar to those of lipiodol and PVA. That is, the black shadow (peripheral vascular networks) disappeared after embolization.

Figure 2B shows reconstructed three-dimensional CT images of renal artery embolization of PGEL, lipiodol, and PVA at different times. Due to its X-ray screening properties, PGEL showed excellent CT imaging capability during the 12-week embolization period. However, after PVA embolization, the kidney was invisible on plain CT images. In addition, although the kidneys were visible in CT images after lipiodol embolization, the deposition of lipiodol in the kidney decreased with time.

Gross photographs showed that renal arterial embolization with PGEL was effective, the embolized kidney uniformly shrank with white calcification, and no failed embolization was found in the left kidney (Fig. 2C). In addition, no abnormal changes in other organs were observed. In contrast, lipiodol had almost no embolic effect on the kidney, and CT re-examination one week after embolization showed recanalization of the renal artery. Moreover, the embolic efficacy of PVA was similar to that of PGEL, and the targeted kidney showed significant atrophy.

Histopathological analysis of the embolized kidney tissues was also performed by hematoxylin and eosin (H&E) staining at week 12 after embolization. The pathological images of the kidney indicated that the renal artery wall was destroyed and PGEL was scattered around the renal artery (Fig. 2D). PGEL embolization-induced ischemic necrosis destroyed the glomeruli, nuclei, and tissues. Similar to the embolic efficacy of PGEL, renal histopathology after PVA embolization also showed extensive necrosis and normal glomeruli and cell nuclear structures were absent. Similar to the control group, the targeted renal tissue pathology showed normal glomeruli and cell nuclear structures after 12 weeks of lipiodol embolization.

### Embolization effect of PGEL on VX2 tumor-bearing rabbits

**Fig. 3:**
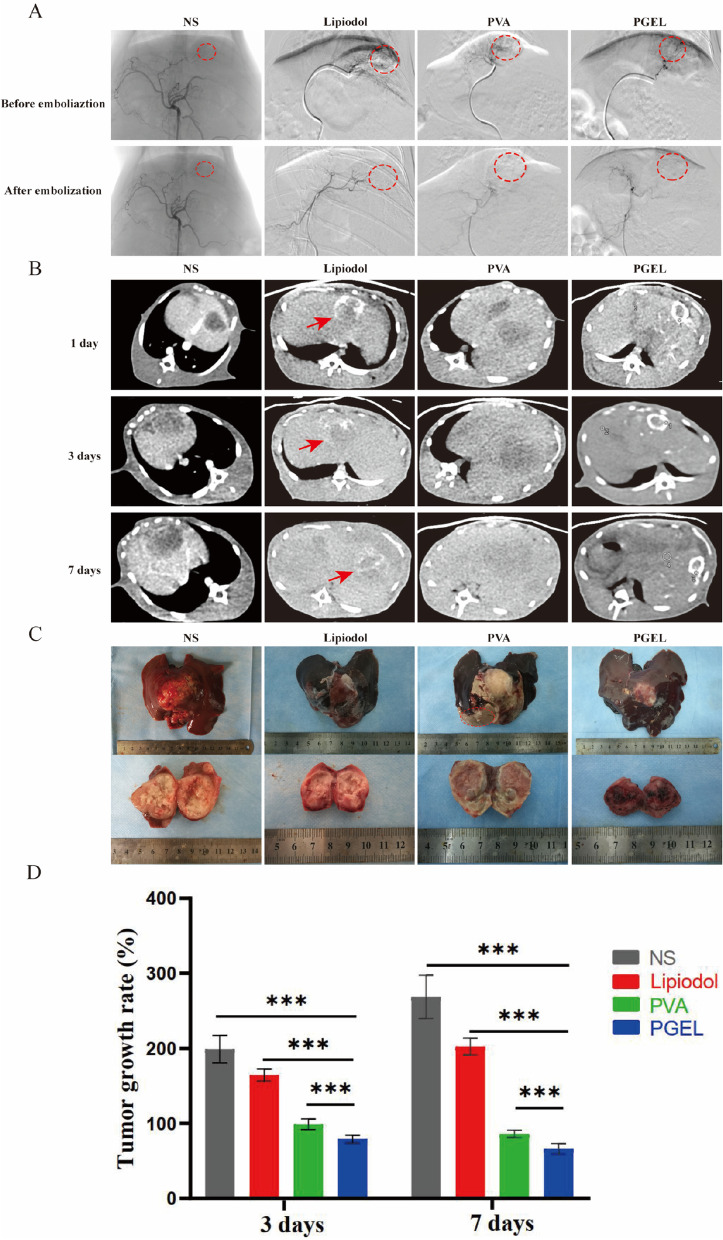
Transcatheter hepatic artery embolization in VX2 tumor-bearing rabbits with lipiodol, PVA, and PGEL. **A** DSA images of tumors before and after embolization. The red dashed circle marks the position of the tumors (before embolization; the tumor vessels were tortuous and increased, and obvious tumor staining could be seen. After embolization, the tumor staining disappeared. **B** CT images at 1, 3, and 7 days after embolization. **C** Gross and sectioned photographs 7 days after embolization. The area within the red dashed circle in gross photos of PVA failed embolization. **D** Tumor growth rate at 3 and 7 days after embolization. *P < 0.05, **P < 0.01, and ***P < 0.001

No difference was found in VX2 tumor angiography before and after NS infusion (Fig. 3A). After TAE of PGEL, lipiodol, or PVA, dark shadows disappeared in digital subtraction angiography (DSA) images (the area surrounded by a red dotted line). CT scans were performed at specific times after treatment (Fig. 3B). Although inferior to PGEL, lipiodol was selectively deposited in the tumor one week after treatment. However, different from PGEL, lipiodol was gradually washed by blood scouring over time. CT images indicated that lipiodol deposition in the tumor decreased, while PGEL was deposited in the tumor for a long time without being washed by blood flow. Gross and sectioned photographs further demonstrated that PGEL was evenly distributed in the tumor tissue and was effective in tumor embolization (Fig. 3C). Failed thrombosis (the area surrounded by red dotted lines) was seen in gross photographs after PVA embolization. Since the lipiodol was scoured by the blood flow, tumor vessel recanalization and neoplasm recurrence were evident.

The antitumor effects of TAE therapy were analyzed in VX2 tumor-bearing rabbits for up to 7 days. On Day 3 after treatment, the PGEL group exhibited the lowest tumor GR compared to the NS, lipiodol, and PVA groups (79.0 ± 5.3% vs. 198.8 ± 18.4% vs. 171.0 ± 8.0% vs. 98.7 ± 7.2%, respectively, *P* < 0.001) (Fig. 3D). Similarly, on Day 7, the tumor GR was the lowest in the PGEL group compared with the NS, lipiodol, and PVA groups (66.0 ± 7.2% vs. 268.6 ± 28.6% vs. 202.4 ± 11.1% vs. 85.9 ± 4.9%, respectively, *P* < 0.001).

**Fig. 4:**
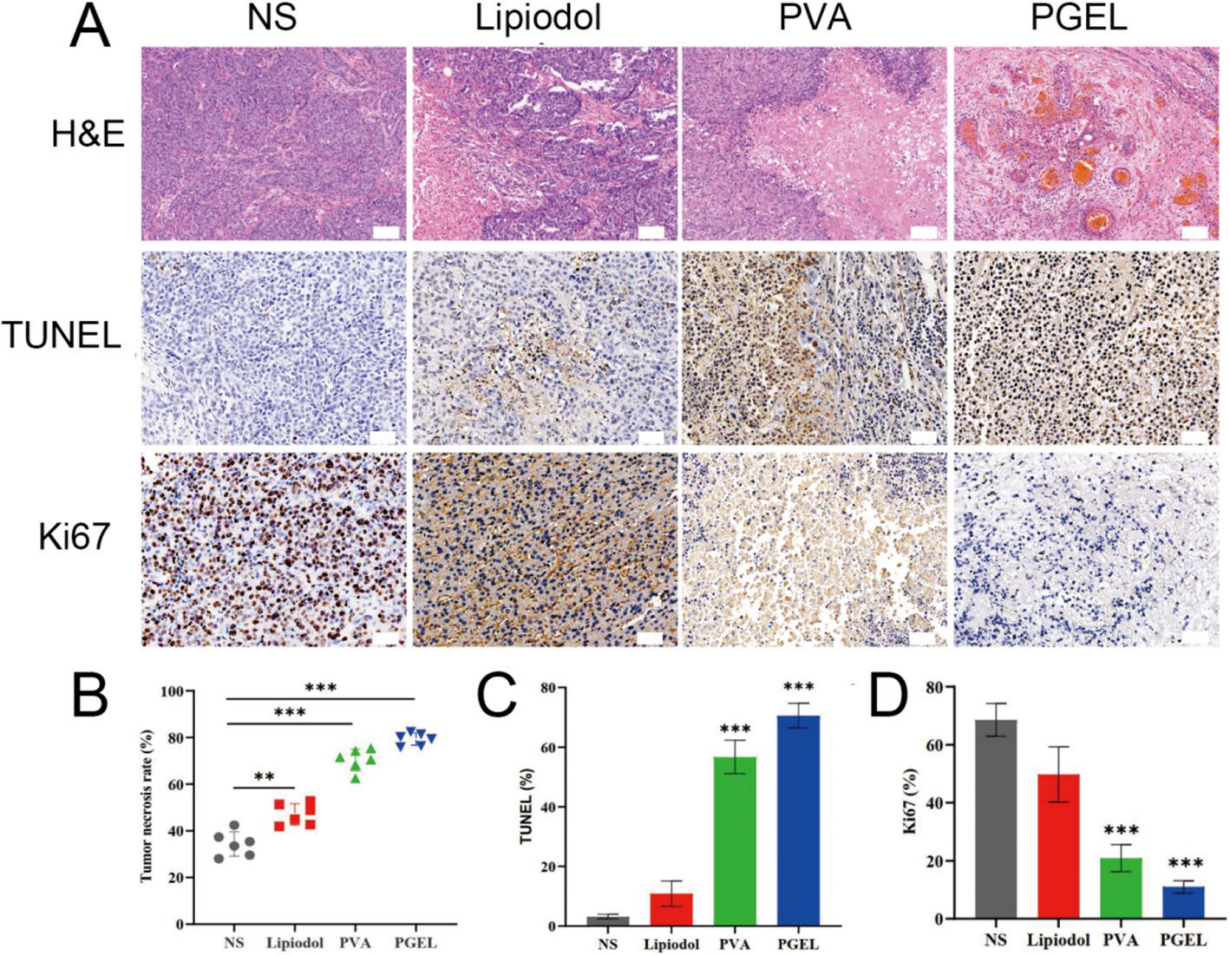
H&E and immunohistochemical (IHC) characterization of tumor necrosis, apoptosis, and proliferation in VX2 tumor-bearing rabbits 7 days after embolization with NS, lipiodol, PVA, and PGEL. **A** Tumor tissue sections were stained with H&E (×100 magnification; the scale bar represents 100 μm), TUNEL (×200 magnification; the scale bar represents 50 μm), and Ki67 (×200 magnification; the scale bar represents 50 μm). **B** Tumor necrosis rate. **C**-**D** Quantitative analysis of TUNEL and Ki67. *P < 0.05, **P < 0.01, and ***P < 0.001

H&E staining confirmed the highest TNR in the PGEL group compared to the NS, lipiodol, and PVA groups (79.3 ± 2.7% vs. 34.4 ± 5.2% vs. 47.1 ± 4.6% vs. 70.4 ± 4.6%, respectively, *P* < 0.01) (Fig. 4A and B). After PGEL embolization, PGEL filled the tumor vessels, indicating that PGEL occluded tumor vessels well (Fig. 4A). In addition, TUNEL staining indicated that the number of apoptotic cells was the highest in the PGEL group compared to the NS, lipiodol, and PVA groups (70.6 ± 4.2% vs. 3.2 ± 0.8% vs. 10.9 ± 4.2 vs. 56.7 ± 5.6%, respectively, *P* < 0.01) (Fig. 4A and C). Correspondingly, immunohistochemical staining of Ki-67, which reflects the proliferation of cells, revealed that proliferation was the lowest in the PGEL group (Fig. 4A and D).

### Evaluation of the postembolization tumor microenvironment

**Fig. 5:**
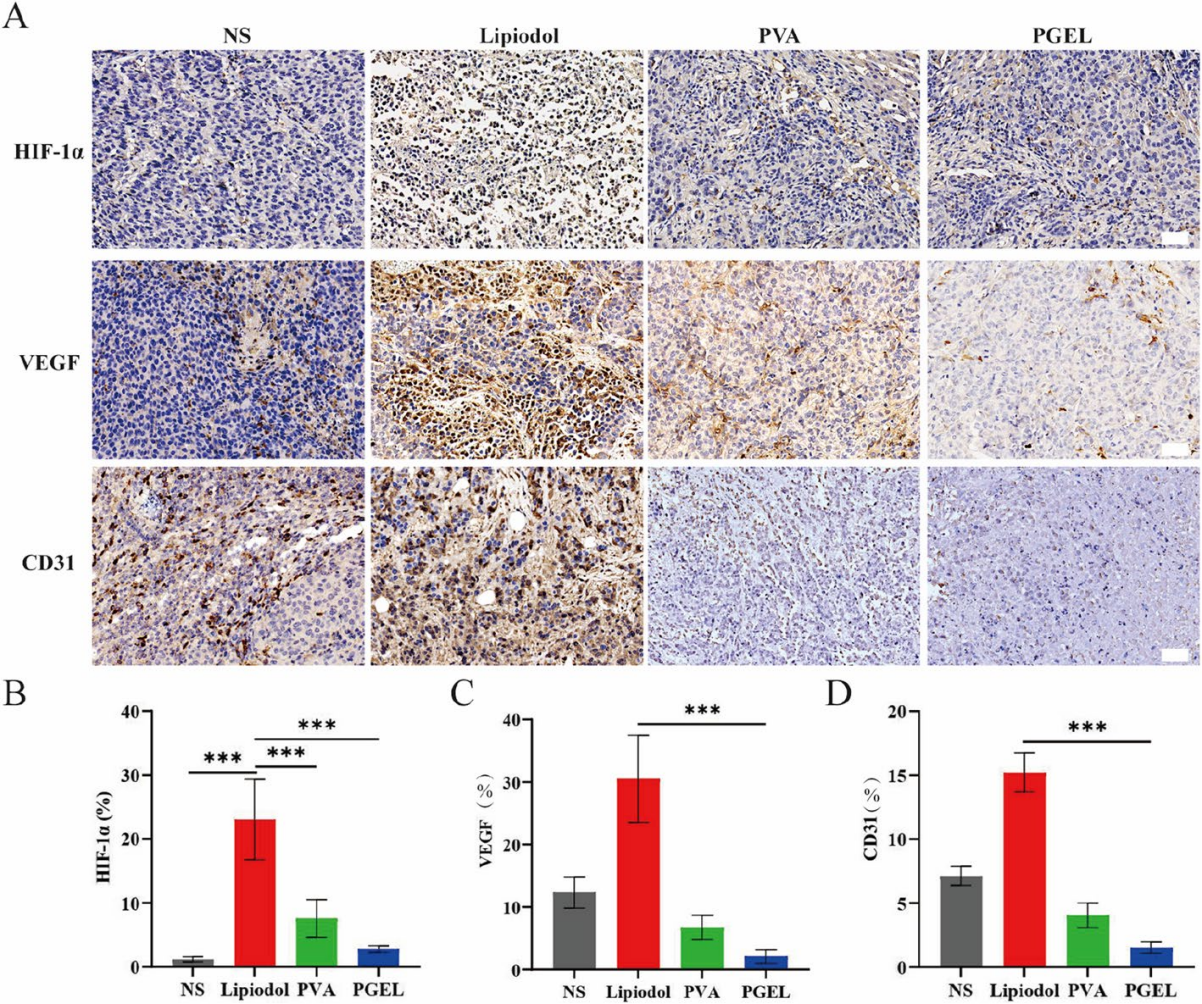
IHC evaluations of the neovascularization of VX2 tumor-bearing rabbits 7 days after embolization with NS, lipiodol, PVA, and PGEL. **A** IHC staining of HIF-1α, VEGF, and CD31 (×200 magnification; the scale bar represents 50 μm). **B**-**D** Expression and quantitative analysis of HIF-1α, VEGF, and CD31.

Three markers (HIF-1α, VEGF, and CD31) were used to assess the tumor microenvironment on Day 7 after TAE therapy (Fig. 5A). Although TAE effectively inhibited tumor growth by cutting off the tumor blood supply, HIF-1α was increased to varying degrees after lipiodol, PVA, and PGEL embolization compared with the corresponding levels in the control group (23.0 ± 6.3% vs. 7.6 ± 3.0% vs. 2.8 ± 0.5% vs. 1.2 ± 0.4%, respectively, *P* < 0.001) (Fig. 5B). Furthermore, the expression levels of VEGF and CD31 were significantly lower in the tumor slices treated with PGEL than in those treated with lipiodol, NS, and PVA (2.1 ± 1.1% vs. 30.5 ± 7.0% vs. 12.3 ± 2.5% vs. 6.8 ± 1.9% for VEGF and 1.5 ± 0.4% vs. 15.2 ± 1.5% vs. 7.1 ± 0.7% vs. 4.0 ± 0.9% for CD31, respectively, all *P* < 0.001) (Fig. 5C and D), further suggesting that PGEL achieved the so-called artery-casting embolization, that is, complete occlusion of all levels of arteries, through its good flowability and embolization.

#### Safety

**Fig. 6:**
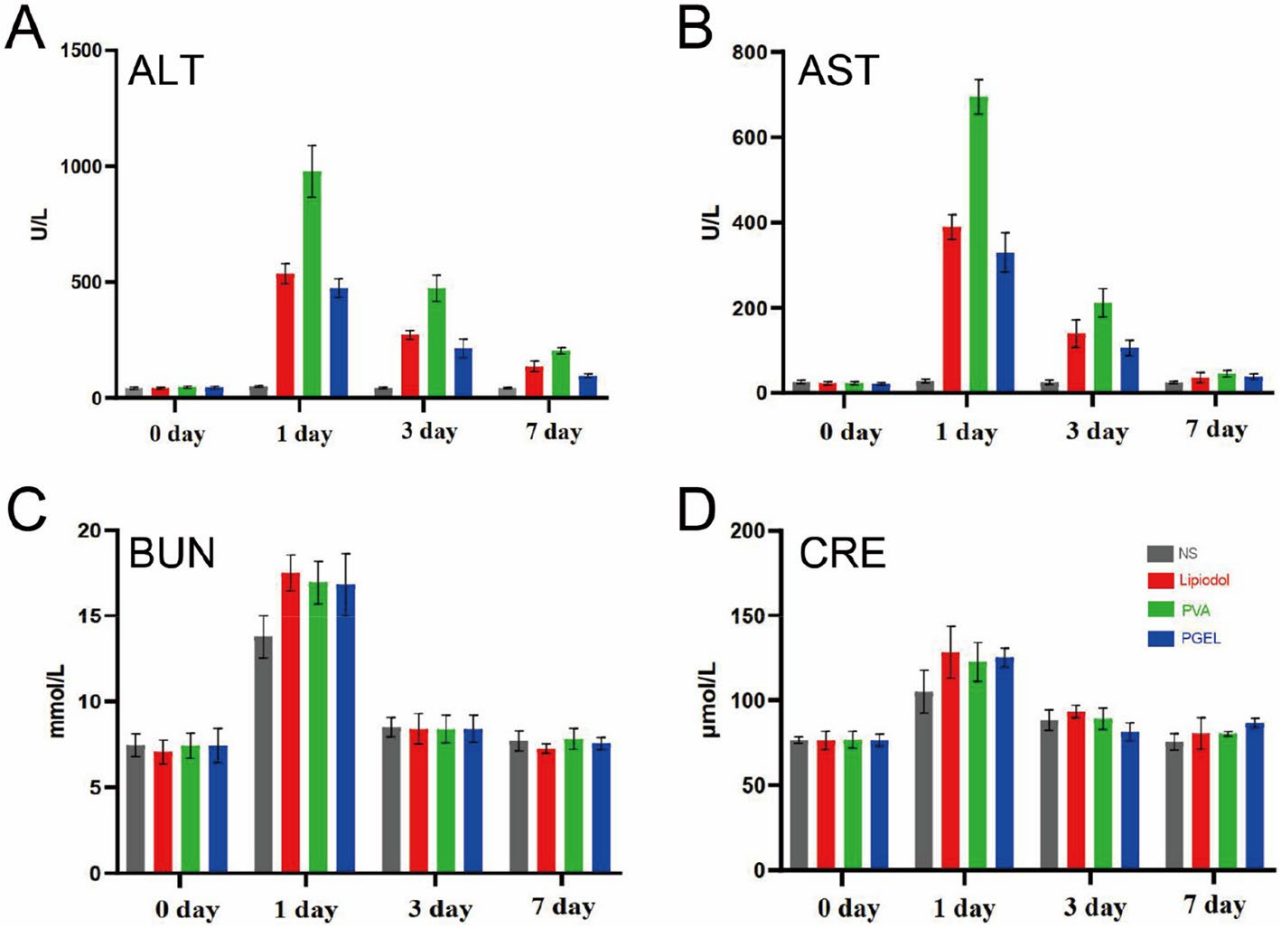
Biocompatibility evaluations of PGEL. **A**-**D** Hepatorenal function of VX2 tumor-bearing rabbits at various time points after treatments: ALT (alanine aminotransferase), AST (aspartate aminotransferase), BUN (blood urea nitrogen), and CRE (creatinine), *n* = 6

The safety of PGEL was measured by the liver and renal function of rabbits on Day 1, 3, and 7 after embolization. ALT and AST levels of rabbits reached maximum values 1 day after treatment with lipiodol, PVA, and PGEL (Fig. 6). However, AST returned to normal levels 7 days after TAE, and ALT also improved significantly. Similarly, Fig. 6A-D showed that PGEL had minimal influence on rabbit renal functions. H&E staining images of major organs harvested from rabbits with hepatic artery embolization for VX2 tumor revealed no noticeable damage in the four groups (Supplemental Fig. 1).

## Discussion

PGEL is a novel liquid embolic agent with radiopaque and thermosensitive properties. The present study indicates the safety and ease of using PGEL as a liquid embolic agent. The presence of infarcts confirms that this material achieves the desired embolic purpose and is similar to commercially available embolic materials [18, 19]. This study demonstrates the possibility of using PGEL as a novel liquid embolic agent for clinical embolization therapy. Our results show that a satisfactory embolization effect can be achieved, with the real-time observation of embolic materials during the embolization procedure.

Generally, embolic agents such as gelatin sponges and PVA are mixed with iodinated contrast media and injected into the target vessel during the embolization procedure. However, due to the rapid diffusion of contrast agents after administration, these contrast agents cannot determine the exact location of the embolic agent deposition [20]. In our study, in contrast to PVA, PGEL was injected into the renal arteries of a normal rabbit without being mixed with a contrast agent. In addition, failed embolization was observed when the tumor feeding artery was embolized with PVA but not with PGEL, further demonstrating the advantage of PGEL’s radiopaque property.

Ethiodized oil first entered medical practice as a therapeutic agent in 1901 [21]. Since the 1980s, ethiodized oil has been widely used in TACE and has been considered as a tumor-seeking drug delivery vehicle to facilitate the intracellular entry of chemotherapeutic agents [22]. Unlike lipioid-based emulsions, PGEL is oil-in-water, which provides better embolization than lipioid-based emulsions. Our previous studies [12, 13] demonstrated that PIB nanogels can achieve excellent peripheral artery embolization, but it is non-radiopaque. Thus, to solve the problems existing in lipiodol and PIB nanogels, PGEL was designed, and its good radiopaque property and thermosensitive sol-gel phase behavior were confirmed.

Deschamps et al. [23] recently reported the preparation of a lipiodol Pickering emulsion loaded with oxaliplatin, which, in contrast to a lipiodol emulsion stabilized by small surfactants, released almost 100% of the drug within 24 h. The lipiodol Pickering emulsion containing oxaliplatin released only 15% of the drug within 24 h. Although the lipiodol Pickering emulsion improved the stability and achieved well-controlled drug release, it also failed to solve the problems of low lipiodol embolization intensity, easy metabolic loss, and tumor recurrence and metastasis. Our study revealed that most tumor tissues in the PGEL group were necrotic, while only a few of them in the lipiodol and NS groups were necrotic. The PGEL group had the highest percentage of apoptotic cells and the lowest percentage of proliferating cells. These data confirmed the ability of PGEL to occlude tumor vessels, leading to the starvation of tumor cells, thereby promoting tumor cell apoptosis and inhibiting their proliferation. In addition, the expressions of CD31 and VEGF (which are positively correlated with tumor angiogenesis) were lowest in the PGEL group, which further indicated that PGEL achieved the so-called artery-casting embolization, that is, complete occlusion of all levels of arteries, through its good flowability and embolization. Therefore, TAE based on the injectable PGEL could effectively block tumor vessels without recanalization and inhibit tumorigenesis.

A previous study demonstrated that the tumor interstitial fluid pressure decreased after embolization, which was conducive to drug penetration in the tumor [24]. If PGEL is loaded with drugs, it may play a role in the slow-release rate of the drug. Furthermore, insufficient TAE therapy could lead to elevated hypoxia levels in tumor tissues, which often leads to tumor angiogenesis and relapse [15, 25]. The present study found that although PGEL can embolize tumors more thoroughly than lipiodol and PVA, HIF-1α expression was still up-regulated in the PGEL group compared with the NS group. Therefore, strategies on how to improve tumor hypoxia after PGEL embolization, such as the use of loading drugs to improve hypoxia, warrant further investigation.

The present study has some limitations. First, only PGEL embolization was performed without chemotherapy drugs or other drugs, which was inconsistent with TACE in clinical application. Therefore, our future studies will focus on PGEL drug loading with chemotherapy drugs or other drugs. Second, the number of animals in each group was relatively small, and the observation time after tumor embolization was relatively short. However, prolonged follow-up could lead to tumor volumes exceeding 10% of body weight, which is not allowed by the Institutional Animal Care and Use Committee. Third, although a large number of preclinical studies have been conducted using the rabbit VX2 model, it is known that the VX2 model has inherent limitations.

## Conclusion

In summary, we developed a novel liquid embolic agent, PGEL, to address problems of poor visibility and traceability of embolic agents during embolization, tumor collateral circulation, and vascular recanalization after TAE treatment, and patient re-examination after TAE treatment. Compared with PVA and lipiodol, PGEL achieved vessel-casting embolization of all levels of arteries in normal rabbits after renal artery embolization. Moreover, PGEL exhibited good CT imaging capability within 12 weeks after embolization. Embolization of VX2 tumor-bearing rabbits indicated that PGEL effectively inhibited tumor collateral circulation and vascular recanalization, and its antitumor effect was superior to that of PVA and lipiodol. Additionally, the vessel-casting embolization effect markedly reduced the expression levels of HIF-1α, VEGF, and CD31, which were often elevated because of the local hypoxia induced by PVA and lipiodol in the TAE treatment. PGEL has good flowability, high-resolution imaging ability, and good biocompatibility, which is beneficial for precise embolization, and could be a potential novel blood-vessel-embolic material for TAE of HCC.

## Supplementary Information

**Additional file 1:** **Supplementary Figure 1. **Representativehistological images of heart (A, B), liver (C, D), spleen (E, F), lung (G, H)and kidney (I, J) of VX2 tumor-bearing rabbits on day 7 after embolization(original magnification, ×100 and ×400. Scale bar is 100μm and 20μm).

## Data Availability

All data generated or analyzed during this study are included in the published article and supplementary files.
