# Supplementary material for: Renal and hepatic artery embolization with Pickering gel emulsion of lipiodol in rabbit
Source: BMC Cancer. 2022 Dec 12;22:1300. doi: 10.1186/s12885-022-10337-5 (PMC9743509; doi:10.1186/s12885-022-10337-5)
Supplement: Supplementary file 1 — Additional file 1: Supplementary Figure 1. Representativehistological images of heart (A, B), liver (C, D), spleen (E, F), lung (G, H)and kidney (I, J) of VX2 tumor-bearing rabbits on day 7 after embolization(original magnification, ×100 and ×400. Scale bar is 100μm and 20μm). [file 12885_2022_10337_MOESM1_ESM.docx]

**[Supplemental Materials](https://pubs.rsna.org/page/radiology/author-instructions#supplemental_material)**


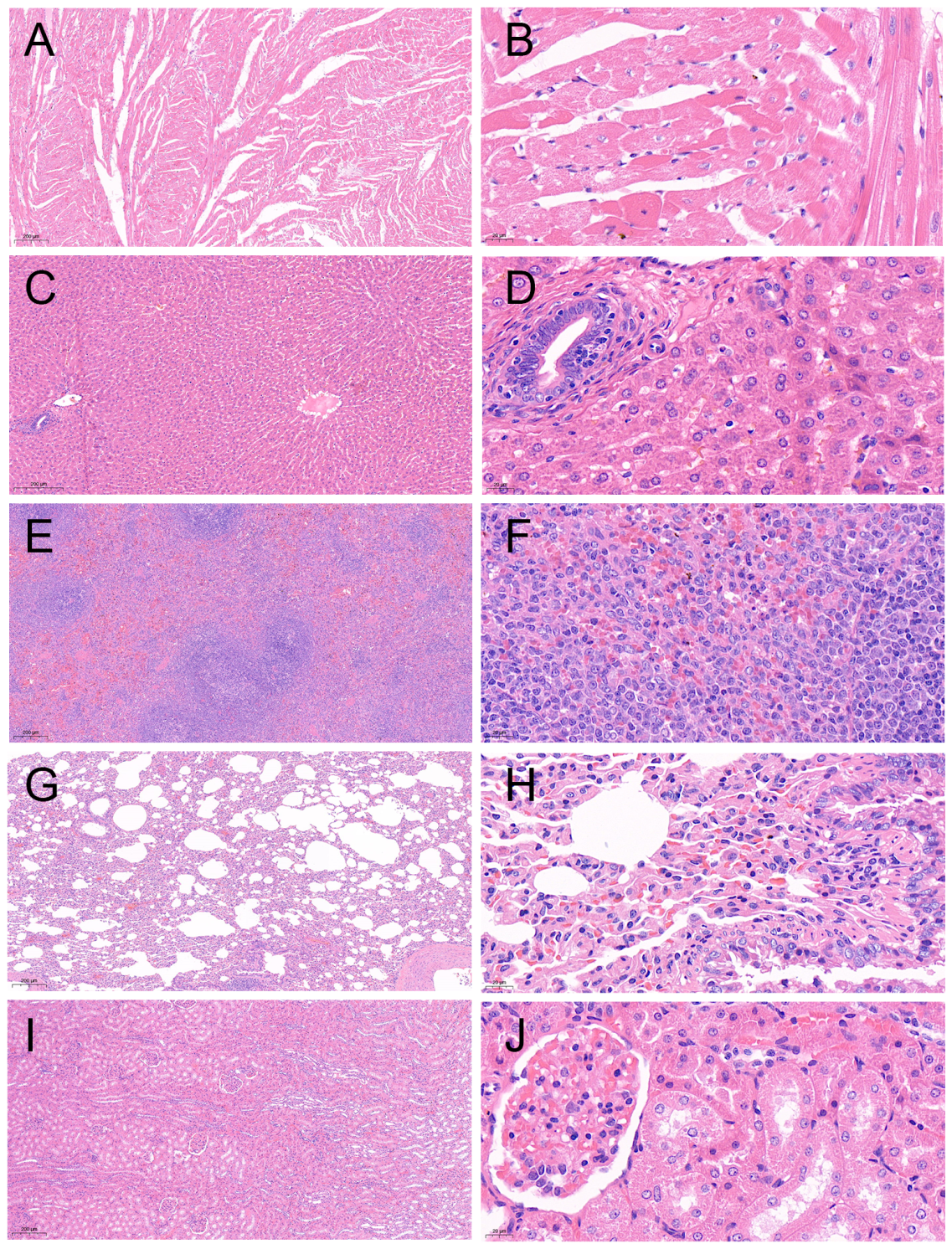


**Supplementary Fig.1**

**Supplementary Figure1** Representative histological images of heart (A, B), liver (C, D), spleen (E, F), lung (G, H) and kidney (I, J) of VX2 tumor-bearing rabbits on day 7 after embolization (original magnification, ×100 and ×400. Scale bar is 100μm and 20μm).
